# Homozygous *LMNA* (p.Arg216His) variant in an Ecuadorian patient with atrial flutter and high-grade atrioventricular block: a case report

**DOI:** 10.3389/fcvm.2026.1967331

**Published:** 2026-10-07

**Authors:** Santiago Cadena-Ullauri, Viviana A. Ruiz-Pozo, Rafael Tamayo-Trujillo, Patricia Guevara-Ramírez, Elius Paz-Cruz, Rita Ibarra-Castillo, José Luis Laso-Bayas, Paul Onofre-Ruiz, Alejandro Cabrera-Andrade, Ana Karina Zambrano

**Affiliations:** 1 Universidad UTE, Facultad de Ciencias de la Salud Eugenio Espejo, Centro de Investigación Genética y Genómica, Quito, Ecuador; 2 Clinical Cardiac Electrophysiologist, Quito, Ecuador; 3 Universidad UTE, Facultad de Ciencias de la Salud Eugenio Espejo, Quito, Ecuador; 4 Grupo de Bio-Quimioinformática, Universidad de Las Américas, Quito, Ecuador; 5 Carrera de Enfermería, Facultad de Ciencias de la Salud, Universidad de Las Américas, Quito, Ecuador

**Keywords:** case report, Ecuador, genetics, genomics, healthcare, laminopathy, *LMNA*

## Abstract

Cardiac laminopathies caused by *LMNA* variants may present with conduction disease, atrial arrhythmias, and ventricular dysfunction, sometimes before overt ventricular dilation. We report a 42-year-old Ecuadorian man with recurrent syncope and progressive exertional dyspnea who developed atrial flutter with a markedly slow ventricular response, high-grade atrioventricular block, and severe bradycardia. Next-generation sequencing using the TruSight Cardio panel identified NM_170707.4(*LMNA*):c.647G > A (p.Arg216His) in a homozygous state. Franklin by QIAGEN classified the variant as pathogenic under the ACMG/AMP framework, although ClinVar contains conflicting interpretations and variant-specific functional and segregation studies were unavailable. Genetic ancestry analysis showed 89% Native American, 8.5% European, and 2.5% African ancestry. To our knowledge, p.Arg216His has not previously been reported in the homozygous state, whereas prior cardiac reports have involved heterozygous or compound heterozygous carriers, highlighting the novelty of this case report. This case expands the clinical and genotypic spectrum associated with LMNA-related cardiac disease and highlights the value of integrating electrophysiological, imaging, genomic, and ancestry data in underrepresented populations.

## Introduction

Atrial flutter is a supraventricular tachyarrhythmia that can compromise hemodynamic stability when associated with a rapid ventricular response (1). In contrast, high-grade atrioventricular (AV) block is an interruption or delay of electrical conduction from the atria to the ventricles (2). The coexistence of atrial flutter and advanced AV conduction disease therefore represents a complex electrical phenotype that may be associated with symptomatic bradycardia, syncope, heart failure, thromboembolic complications, and, depending on the clinical context, the need for permanent cardiac pacing (3, 4).

The *LMNA* gene encodes lamins A and C, structural and regulatory components of the nuclear lamina that contribute to nuclear integrity, mechanotransduction, chromatin organization, and regulation of gene expression (5, 6). Hundreds of disease-associated *LMNA* variants have been associated with a heterogeneous group of disorders termed laminopathies, which can affect different tissues, including the heart (7). Cardiac involvement may include sinus-node dysfunction, AV conduction disease, atrial and ventricular arrhythmias, and systolic dysfunction, which can occur individually or in combination and may precede the development of an overt dilated or non-dilated cardiomyopathy phenotype (5, 6).

Among reported *LMNA* variants, p.Arg216His affects a residue for which other amino acid substitutions have been associated with cardiac disease (8–13). The p.Arg216His variant itself has been reported in heterozygous individuals with phenotypes including dilated cardiomyopathy and arrhythmias, although its clinical classification remains disputed and variant-specific functional evidence is limited (13, 14).

Latin American populations remain underrepresented in the genomic reference datasets, potentially complicating population-frequency assessment and interpretation of rare variants. Genetic ancestry analysis can provide relevant population context in admixed individuals and may improve interpretation of population allele frequencies; however, global ancestry proportions cannot establish the ancestral origin or pathogenicity of a specific variant (15).

The present case describes an Ecuadorian patient with atrial flutter, high-grade AV block, left atrial dilation, and mildly reduced left ventricular systolic function without ventricular dilation. Clinical, genomic, and ancestry findings were integrated to characterize the phenotype and examine the potential relationship between the *LMNA* p.Arg216His variant and the observed cardiac manifestations.

## Case description

### Patient information and clinical history

A 42-year-old Ecuadorian man, born and residing in Guano, Ecuador, was evaluated for recurrent episodes of syncope and progressive exertional dyspnea. He denied tobacco use, alcohol consumption, and recreational drug use. Physical examination showed a hemodynamically stable patient with blood pressure within the normal range and a body mass index of 24.8 kg/m^2^. Heart rate fluctuated between 35 and 70 beats/min, with documented episodes of bradycardia. Cardiac auscultation revealed a regular rhythm without murmurs or other relevant findings. Laboratory assessment during the most recent hospitalization showed HbA1c 6.07%, total cholesterol 199, LDL cholesterol 134.70, HDL cholesterol 39.10, triglycerides 126, creatinine 1.17–1.20, TSH 2.88, INR 1.18, troponin 0.01, and AST 45.6. Hemoglobin and hematocrit were 18.3 and 54.4, respectively. Serum sodium was initially 132.8 and subsequently 137.8–138.5. His previous medical history included biliary pancreatitis managed conservatively, non-obstructive nephrolithiasis managed clinically, and non-alcoholic fatty liver disease considered to have a low risk of fibrosis and managed through lifestyle modification; confirmatory laboratory or imaging documentation regarding the latter diagnosis was not available.

Family history was notable for a maternal male cousin who reportedly died at 27 years of age from congestive heart failure (Supplementary Figure S1). No medical records or additional clinical information were available to establish the etiology of the heart failure, and no documented history of sudden cardiac death (SCD) was identified. Cascade genetic testing and segregation analysis were not completed because family members declined participation, and no familial DNA samples were available. Consequently, familial segregation, penetrance, and expressivity of the p.Arg216His variant could not be assessed.

### Cardiovascular diagnostic assessment

Transthoracic echocardiogram performed on July 18, 2025, showed a left ventricular ejection fraction (LVEF) of 60.4%. The indexed left atrial and right atrial volumes were 35.7 and 31.7 mL/m^2^, respectively. The inferior vena cava measured 22.4 mm, with a reported inspiratory collapse to 12.8 mm (42.8%). The original echocardiographic report recorded a diagnostic conclusion of dilated cardiomyopathy.

In February 2026, a 12-lead electrocardiogram demonstrated atrial flutter with a markedly slow ventricular response of approximately 20 beats/min. The frontal QRS axis was reported at +120°, consistent with right-axis deviation. Complete right bundle branch block was also described, together with aberrant conduction. These findings established the presence of a severe bradyarrhythmic phenotype in the setting of an atrial tachyarrhythmia. During the same month, a transthoracic echocardiography demonstrated a left ventricle with normal dimensions but mildly reduced systolic function, with an LVEF of 47%. The indexed left atrial volume was 35.9 mL/m^2^ and the indexed right atrial volume was 31.9 mL/m^2^. The inferior vena cava measured 24.4 mm, with inspiratory reduction to 12.8 mm and a reported collapse of 47.7%. A timeline with the most relevant episodes of care is illustrated in Figure 1.

**Figure 1:**
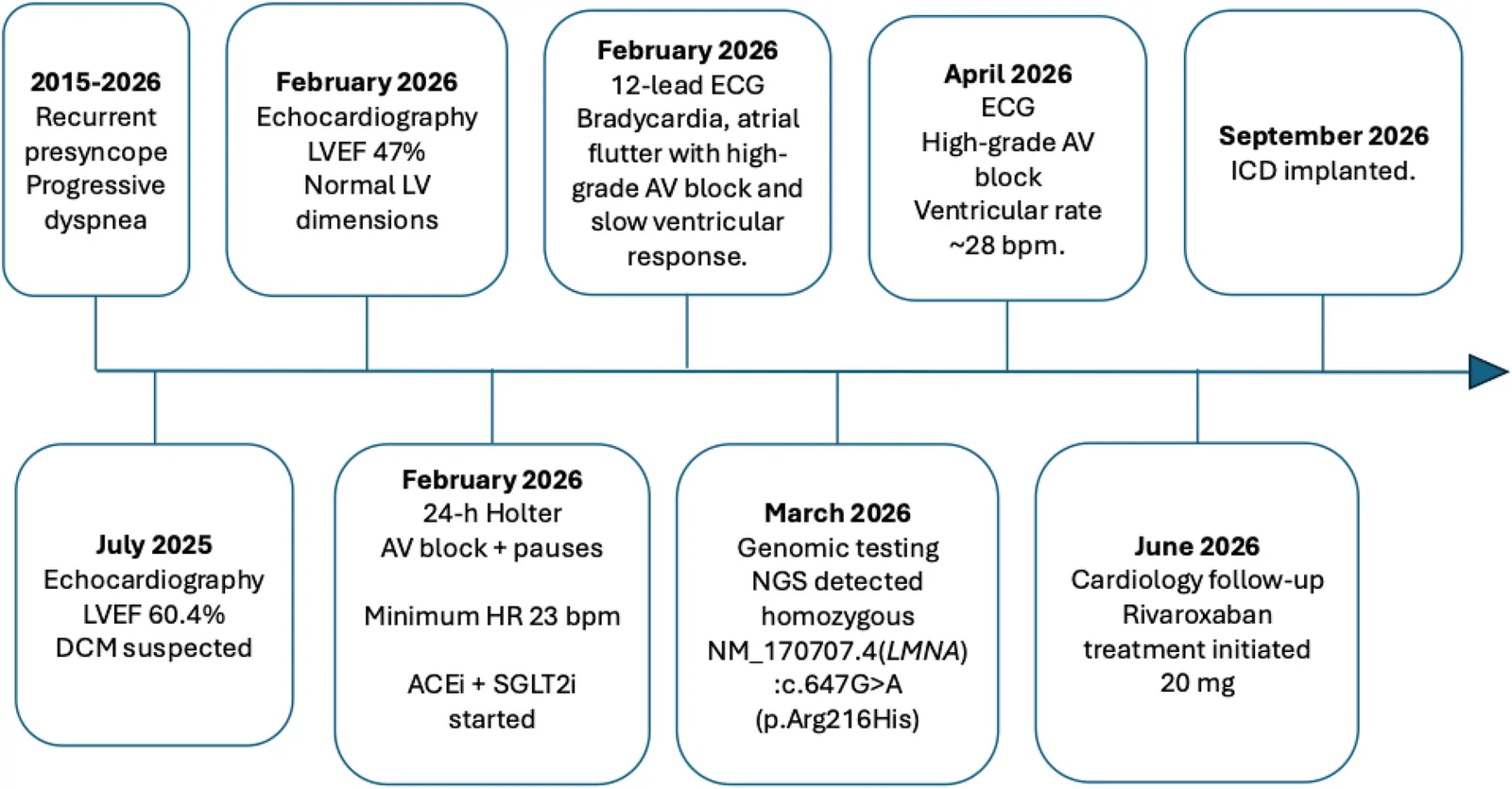
Timeline of the clinical, electrophysiological, therapeutic, and genomic findings. The timeline summarizes the progression from recurrent presyncope and dyspnea to atrial flutter with severe bradycardia and AV conduction disease, mild LV systolic dysfunction, identification of the homozygous NM_170707.4(LMNA):c.647G>A (p.Arg216His) variant, initiation of rivaroxaban 20 mg/day in June 2026, and subsequent ICD implantation in September 2026. Abbreviations: ACEi, angiotensin-converting enzyme inhibitor; AV, atrioventricular; bpm, beats per minute; DCM, dilated cardiomyopathy; ECG, electrocardiogram; HR, heart rate; ICD, implantable cardioverter-defibrillator; LVEF, left ventricular ejection fraction; LV, left ventricle; NGS, next-generation sequencing; SGLT2i, sodium-glucose cotransporter-2 inhibitor.

Twenty-four-hour Holter monitoring performed during the same month documented predominantly sinus rhythm, with no symptoms recorded during monitoring and no episodes of atrial fibrillation. The maximum, minimum, and mean heart rates were 78, 23, and 42 beats/min, respectively. A total of 4,856 supraventricular ectopic beats were reported, including 1,208 isolated ectopic beats, 744 couplets, and 585 episodes of supraventricular tachycardia. No ventricular ectopic beats were recorded. First-degree AV block was present, together with episodes of second-degree AV block described as Mobitz type I. The recording also identified 6,265 atrioventricular pauses exceeding 2 s, with a maximum reported duration of 2.644 s.

A subsequent ECG obtained in April 2026 demonstrated a severely bradycardic ventricular response, with a ventricular rate of approximately 28 beats/min and prolonged pauses. The atrial rhythm was described as irregular, without clearly discernible sinus P waves and with undulating atrial activity most evident in leads II, III, aVF, and V1. QRS complexes were narrow and showed preserved morphology, and no acute ischemic ST-segment or T-wave abnormalities were described. The final clinical interpretation of the tracing was atrial flutter associated with high-grade AV block and an extremely slow ventricular response.

During the initial hospitalization, the patient remained under clinical observation, and no permanent cardiac device was implanted. The available clinical records did not specify the rationale for initially deferring device therapy. Low-dose angiotensin-converting enzyme inhibitor and sodium-glucose cotransporter-2 inhibitor therapy was initiated to optimize ventricular function, with a favorable short-term clinical response. During subsequent follow-up, rivaroxaban 20 mg once daily was initiated in June 2026. Following continued cardiology and electrophysiology evaluation, an ICD was implanted in September 2026. No cardioversion or catheter ablation was documented.

The timeline summarizes the progression from recurrent presyncope and dyspnea to atrial flutter with severe bradycardia and AV conduction disease, mild LV systolic dysfunction, identification of the homozygous NM_170707.4(LMNA):c.647G > A (p.Arg216His) variant, initiation of rivaroxaban 20 mg/day in June 2026, and subsequent ICD implantation in September 2026. Abbreviations: ACEi, angiotensin-converting enzyme inhibitor; AV, atrioventricular; bpm, beats per minute; DCM, dilated cardiomyopathy; ECG, electrocardiogram; HR, heart rate; ICD, implantable cardioverter-defibrillator; LVEF, left ventricular ejection fraction; LV, left ventricle; NGS, next-generation sequencing; SGLT2i, sodium-glucose cotransporter-2 inhibitor.

### Genomic assessment

Given the combination of recurrent syncope, severe conduction-system disease, atrial arrhythmia, and ventricular systolic dysfunction, genomic testing for inherited cardiac disease was performed. Peripheral blood was collected in EDTA tubes, and genomic DNA was extracted using the PureLink Genomic DNA Mini Kit (Invitrogen, Thermo Fisher Scientific). DNA concentration was assessed using Qubit fluorometric quantification. Sequencing libraries were prepared with the TruSight Cardio Sequencing Kit (Illumina) and sequenced on the MiSeq platform. Sequence reads were processed and aligned to the GRCh38/hg38 human reference genome using the DRAGEN Enrichment pipeline in BaseSpace Sequence Hub, and variants were annotated using the Ensembl Variant Effect Predictor. Clinical variant interpretation was subsequently performed using Franklin by QIAGEN according to the ACMG/AMP framework (16, 17).

NGS identified the missense variant NM_170707.4(*LMNA*):c.647G > A (p.Arg216His) in a homozygous state (Figure 2). The homozygous *LMNA* variant was identified using the TruSight Cardio panel. The variant call passed all quality filters and was supported by a total site depth of 86 reads. All genotype-level reads supported the alternative allele (Allele Depth=0/84), resulting in an allele fraction (AF) of 1.00. The Phred-scaled genotype likelihoods (PL = 83,53,0), were consistent with the homozygous alternative genotype, which showed the lowest PL value of 0, while the genotype quality was (GQ = 44), provided additional support for the 1/1 genotype call. Segregation analysis could not be performed because family members declined participation, and no additional familial samples were therefore available for genetic testing. Franklin by QIAGEN classified the variant as pathogenic based on ACMG/AMP criteria PS4, PM1, PP2, PM2, PP3, and PM5. Because the variant has conflicting interpretations in external clinical databases and no segregation or variant-specific functional studies were available in this patient, the molecular result was interpreted in conjunction with the clinical phenotype rather than as independent evidence of causality.

**Figure 2:**
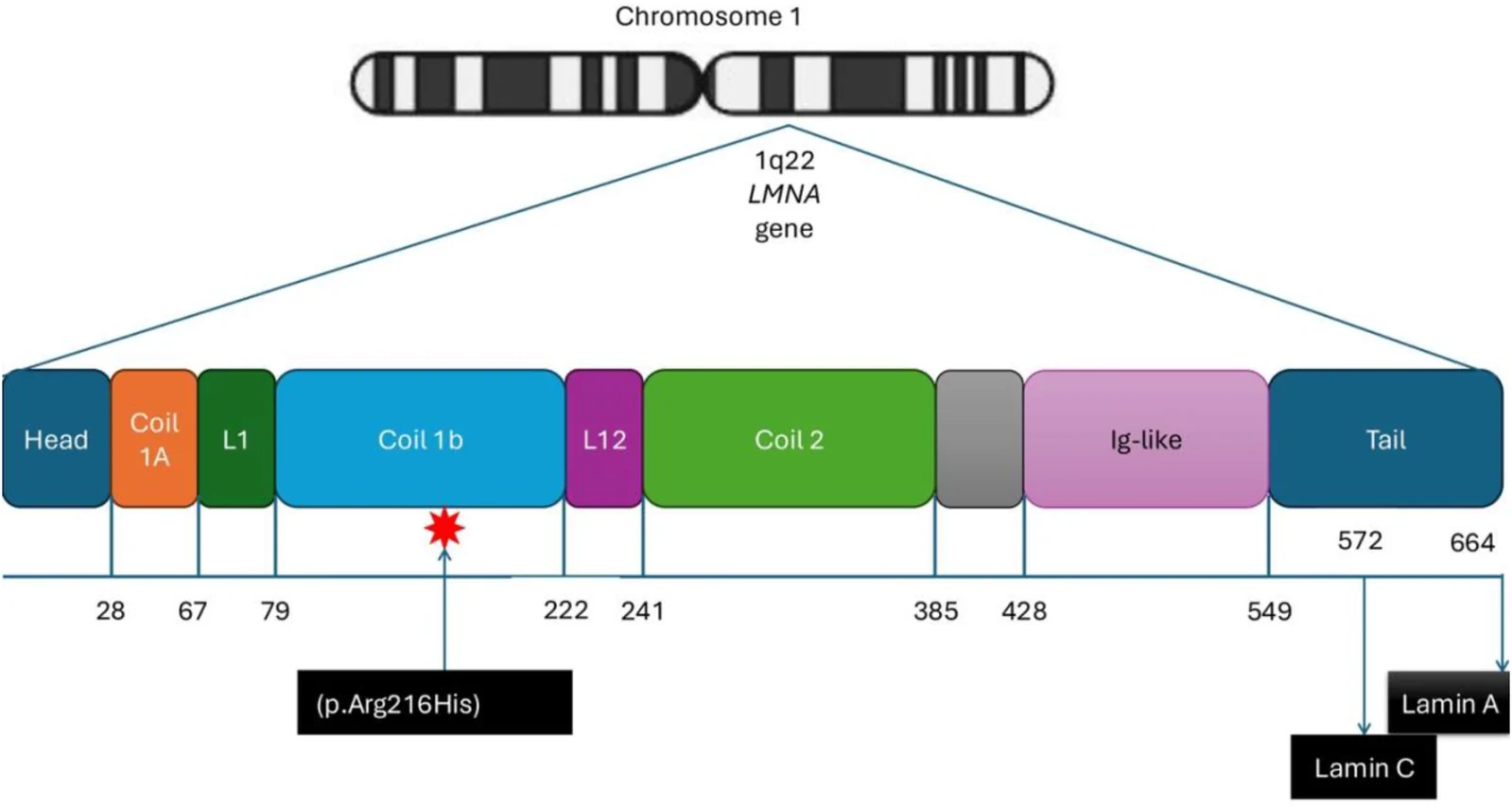
Schematic representation of lamin A/C and localization of the p.Arg216His variant. LMNA is located at chromosome 1q22 and encodes lamin A and lamin C. The schematic depicts the principal structural regions of lamin A/C, including the N-terminal head, coil 1A, linker regions, coil 1B, coil 2, immunoglobulin-like domain, and C-terminal tail. The p.Arg216His substitution is located within the C-terminal portion of the coil 1B region of the central *α*-helical rod domain, which contributes to lamin dimerization and higher-order assembly. The position of Arg216 is indicated in red (5, 18, 19).

*LMNA* is located at chromosome 1q22 and encodes lamin A and lamin C. The schematic depicts the principal structural regions of lamin A/C, including the N-terminal head, coil 1A, linker regions, coil 1B, coil 2, immunoglobulin-like domain, and C-terminal tail. The p.Arg216His substitution is located within the C-terminal portion of the coil 1B region of the central *α*-helical rod domain, which contributes to lamin dimerization and higher-order assembly. The position of Arg216 is indicated in red (5, 18, 19).

### Genetic ancestry analysis

To characterize the patient's population-genetic background, ancestry was estimated using a multiplex panel of 46 ancestry-informative insertion/deletion markers (AIM-InDels) according to Zambrano, A et al. (15). Individual admixture proportions were inferred using the Bayesian clustering algorithm implemented in STRUCTURE v2.3.4 with Native American, European, and African reference populations. The analysis demonstrated a predominantly Native American ancestry component of 89%, followed by European ancestry of 8.5% and African ancestry of 2.5%. These findings documented the patient's admixed Ecuadorian genomic background and were considered complementary population context for interpretation of the genetic findings.

## Discussion

This case describes an Ecuadorian patient with atrial flutter, high-grade AV block, left atrial dilation, and mild left ventricular systolic dysfunction without ventricular dilation. Furthermore, NGS allowed the identification of a homozygous NM_170707.4(*LMNA*):c.647G > A (p.Arg216His) variant. The combination of atrial arrhythmia, advanced conduction-system disease, and early ventricular dysfunction is compatible with the spectrum of cardiac laminopathies. One of the main contributions of this case lies in the NGS-detected homozygous genotype, as previously reported affected carriers of p.Arg216His have been described in heterozygosity.

*LMNA* encodes the nuclear intermediate filament proteins lamin A and lamin C, which are generated through alternative splicing and collectively referred to as lamin A/C (5, 20–22). These proteins are widely expressed in differentiated somatic cells and constitute major components of the nuclear lamina. In addition to providing structural support to the nuclear envelope, lamin A/C contributes to chromatin organization, transcriptional regulation, cell-cycle control, and DNA replication and repair through interactions with chromatin and other nuclear proteins (5, 20, 22–24). Pathogenic *LMNA* variants cause a heterogeneous group of disorders collectively termed laminopathies, which may involve the cardiovascular, neuromuscular, metabolic, and skeletal systems (5, 25, 26).

In cardiac tissue, lamin A/C contributes to nuclear stability, cardiomyocyte differentiation, cardiac development, mechanotransduction, and resistance to mechanical stress (22–24). Altered lamin A/C function may disrupt nuclear architecture and cellular signaling in cardiomyocytes and specialized conduction-system cells. Accordingly, *LMNA*-related cardiac disease commonly combines myocardial dysfunction with electrical abnormalities, including sinus-node dysfunction, atrioventricular conduction disease, atrial tachyarrhythmias, and ventricular arrhythmias (21, 27–29). The extent and nature of cardiomyocyte involvement may vary according to the type and functional consequence of the *LMNA* variant, particularly its effects on nuclear structure, cellular mechanics, and signaling pathways. This genotype-dependent variability is clinically relevant because it may influence disease expression, progression, and risk stratification (29). In addition, *LMNA*-related cardiomyopathies have been associated with a higher risk of heart-failure-related death and a less favorable response to standard therapy than *TTN*-related cardiomyopathies, supporting the prognostic importance of distinguishing between these genetic etiologies (20, 30).

The identified variant, NM_170707.4(*LMNA*):c.647G > A (p.Arg216His), is a missense substitution that replaces arginine with histidine at residue 216 of lamin A/C. Variant analysis and interpretation were performed using Franklin by QIAGEN, a platform for genomic variant analysis and clinical interpretation (16). Franklin classified the variant as pathogenic according to the ACMG/AMP criteria PS4, PM1, PP2, PM2, PP3, and PM5. However, the aggregate ClinVar classification remains “Conflicting classifications of pathogenicity”; notably, the most recent submission, from January 2026, classifies the variant as Likely Pathogenic (13). Therefore, the pathogenic classification reported in this study should be understood as the result of the interpretive framework implemented in Franklin by QIAGEN and the evidence available at the time of analysis, rather than as an externally established consensus.

PS4 was applied because p.Arg216His has previously been identified in individuals with cardiac disease. In one report, a 63-year-old carrier was diagnosed with dilated cardiomyopathy, and fibroblast nuclear morphology analysis identified 15.67% abnormal nuclei (13, 14). PM1 was assigned because the variant lies within a region considered functionally relevant and enriched for pathogenic or likely pathogenic changes, including a 171-bp interval surrounding the variant (16). PP2 was applied because missense variation is an established disease mechanism in *LMNA* and the surrounding region shows intolerance to genetic variation, with a reported regional constraint z-score of 5.33 (16, 31). PM2 was supported by the variant's very low frequency in population databases (16, 31). In gnomAD v4.1.1, the total allele frequency of p.Arg216His was 0.00004151. The variant was not observed in the Admixed American population (allele frequency = 0) (31). In ALFA (release 20260205170148), the overall allele frequency was 0.000021, while the Latin American 1 and Latin American 2 populations both showed an allele frequency of 0 (32). No homozygous individuals carrying the variant were reported in these databases (31, 32). In ExAC v0.3, the global allele frequency was 0.000008 (33). These data confirm the extreme rarity of the variant in the general population and its absence from the available Latin American/admixed American reference subsets.

Computational evidence further supported a PP3 criterion, as multiple *in silico* prediction tools indicated a potentially deleterious effect of p.Arg216His. REVEL yielded a score of 0.78, corresponding to moderate evidence for a deleterious effect (34), whereas PrimateAI generated a score of 0.81, supporting a deleterious prediction (35). BayesDel produced a score of 0.15, also interpreted as supporting evidence of deleteriousness (36). Consistent predictions were obtained with MutationTaster, DANN, and MetaLR, all of which classified the variant as deleterious (13, 31, 37–40). Furthermore, the CADD is 29.9, highlighting the potential pathogenicity of the variant (31, 41). Taken together, these concordant computational predictions supported the assignment of PP3 under the ACMG/AMP framework. PM5 was supported by the alternative missense substitution *LMNA* c.646C > T, p.(Arg216Cys), which affects the same Arg216 residue. p.(Arg216Cys) has been reported in individuals with dilated cardiomyopathy, atrioventricular conduction disease, and other laminopathy-associated cardiac phenotypes and has been classified as pathogenic or likely pathogenic (8, 10, 11). However, this cannot be directly extrapolated to p.Arg216His, as different amino acid substitutions may have distinct structural and functional consequences.

Liu et al. reported that another variant affecting residue 216 was associated with irregular nuclear morphology, altered lamin A/C localization, perinuclear protein aggregation, and increased apoptosis under environmental stress (8). These findings provide mechanistic support for the functional relevance of codon 216 but do not constitute direct functional evidence for p.Arg216His. The absence of variant-specific functional studies should therefore remain explicit when discussing pathogenicity.

Residue 216 is located within the C-terminal portion of coil 1B in the central *α*-helical rod domain of lamin A/C. Structural analysis of human lamin A/C has shown that coil 1B extends approximately from residues 79 to 222, with residues 204–222 forming a C-terminal hendecad-repeat, untwisted *α*-helical segment that participates in the structural organization of the rod domain (19, 42). Coil 1B contributes to lamin dimer and higher-order assembly: structural studies have demonstrated that this region participates in antiparallel interactions between lamin dimers during A11 tetramer formation, whereas biophysical studies have shown that the 1B domain can self-assemble into lamin-like networks and contributes to the mechanical properties of lamin A (43, 44).

The physicochemical properties of the amino acids involved provide a plausible structural basis for an effect on lamin A/C. Arginine is a polar, basic amino acid whose guanidinium group remains positively charged under physiological conditions, enabling stable electrostatic and hydrogen-bond interactions (45). Histidine contains an imidazole side chain that may alternate between neutral and positively charged states depending on the local pH and protein microenvironment (46). Thus, replacement of arginine by histidine could alter local electrostatic interactions, hydrogen-bonding patterns, and side-chain geometry because arginine provides a persistently positively charged guanidinium group, whereas histidine contains an imidazole group whose protonation state is more sensitive to the local environment. These physicochemical differences may modify local rod-domain stability or intermolecular interactions involved in lamin assembly. However, these proposed consequences remain mechanistic hypotheses because no variant-specific functional experiments have directly evaluated the effects of p.Arg216His on lamin A/C structure, localization, assembly, or cellular function (13, 16).

The homozygosity identified in this patient represents the most notable molecular aspect of the case, given that previous clinically affected carriers of p.Arg216His have been reported in heterozygosity (13, 14, 47). This finding raises the hypothesis that an increased allelic burden could modify disease expression, as patients carrying mutations in cardiac disease-associated genes have shown increased disease severity (48, 49). However, this cannot be demonstrated from a single patient, and further research is required to fully understand the impact of the variant and its zygosity in cardiovascular diseases.

The potential relevance of the homozygous genotype was assessed by comparing the present phenotype with previously reported carriers of *LMNA* p.Arg216His. To our knowledge, no previously published report has described p.Arg216His in the homozygous state; available cardiac reports involve heterozygous carriers or, in one case, a compound heterozygous p.Arg216His/p.Arg331Leu genotype (14, 47, 50, 51). In the study by van Tienen et al., the p.Arg216His variant was identified in heterozygosity in a 63-year-old man with DCM, although individual data regarding atrioventricular conduction abnormalities or arrhythmias were not reported (14). Ferradini et al. described a 56-year-old woman carrying compound heterozygous p.Arg216His/p.Arg331Leu variants in *LMNA* who presented with severe left ventricular systolic dysfunction (LVEF 30%), ventricular and supraventricular arrhythmias, and multiple appropriate implantable cardioverter-defibrillator shocks (47). In comparison, our patient was symptomatic at 42 years of age and exhibited a predominantly electrical phenotype, with atrial flutter, severe bradycardia, first- and second-degree AV conduction abnormalities, high-grade AV block, and recurrent prolonged pauses. Ventricular involvement was comparatively mild, with an LVEF of 47% and normal left ventricular dimensions, and no ventricular arrhythmias were documented. Therefore, these observations do not suggest a strong association between homozygosity and overall disease severity, as the compound heterozygous patient showed more severe ventricular disease (47). Instead, the present findings could be interpreted as a possibility that homozygosity for p.Arg216His may be associated with an earlier or more conduction-predominant phenotype (49, 52, 53). This hypothesis requires further research because individual-level clinical data are incomplete for several previously reported carriers and no segregation or variant-specific mechanistic studies are available.

Risk stratification for SCD has been specifically refined in patients with *LMNA*-related cardiomyopathy (54). Across multicenter studies, NSVT, reduced LVEF, male sex, non-missense variants, and AV block have emerged as important predictors of ventricular arrhythmic risk in *LMNA* variant carriers (55–57). These findings led to the recommendation that in patients with an estimated 5-year risk ≥10% and a manifest cardiac phenotype, including NSVT, LVEF <50%, or AV conduction delay, primary-prevention ICD implantation should be considered (54).

In the present patient, several clinically relevant risk features were present, including male sex, symptomatic high-grade AV conduction disease, and mildly reduced LVEF (47%), and a p.Arg216His variant. These findings support the importance of repeated arrhythmic risk assessment. Although management was initially conservative, continued cardiology and electrophysiology follow-up ultimately led to ICD implantation in September 2026. This clinical evolution illustrates the value of longitudinal reassessment in LMNA-related cardiac disease.

The absence of segregation and functional studies limits conclusions regarding inheritance, pathogenic mechanism, and a possible gene-dosage effect. These limitations do not, however, invalidate the molecular findings presented. The variant was identified using a comprehensive NGS workflow incorporating sequencing quality control, read alignment, variant calling, annotation, population-frequency assessment, clinical-database review, computational prediction, and phenotype-oriented interpretation. In validated assays, high-quality single-nucleotide variants may be reported without routine Sanger confirmation when sequencing quality metrics are satisfied (58, 59).

The ancestry analysis adds relevant population context because Ecuadorian and other admixed Latin American populations remain comparatively underrepresented in clinical genomic datasets. The patient's ancestry composition of 89% Native American, 8.5% European, and 2.5% African ancestry, reflects the admixed genetic background characteristic of many Ecuadorian populations (5, 15, 60–65). This information may be useful when contextualizing population allele frequencies and emphasizes the value of reporting cardiogenetic findings from historically underrepresented groups (66–68). Nevertheless, global ancestry proportions cannot determine the ancestral origin of the p.Arg216His alleles, demonstrate identity by descent, establish parental relatedness, or support a founder effect. The ancestry result should therefore be interpreted as complementary genomic information rather than evidence supporting variant pathogenicity or homozygosity.

In conclusion, this article integrates a clinical, genomic and ancestry characterization to provide a comprehensive overview of the disease. The phenotype is compatible with cardiac laminopathy, and the homozygosity of the p.Arg216His variant is an unusual observation that may expand the reported genotypic spectrum of *LMNA*-associated cardiac disease. However, conflicting external variant classifications and the absence of segregation and variant-specific functional studies preclude definitive conclusions regarding causality, inheritance, or a dosage-dependent effect. Additional independently characterized cases will be required to clarify the clinical significance of p.Arg216His in the homozygous state.

## Data Availability

The data presented in the study are deposited in the Sequence Read Archive from National Center of Biotechnology Information, BioSample accession number PRJNA1534577.

## Supplementary material

Supplementary Figure S1 Pedigree of the proband's family. The proband (arrow) is a male with atrial flutter and atrioventricular block. A maternal male cousin died from congestive heart failure at 27 years of age. Squares indicate males, circles females, and diagonal lines deceased individuals. Colored symbols indicate the documented cardiac phenotypes according to the legend.
