# Supplementary material for: Homozygous LMNA (p.Arg216His) variant in an Ecuadorian patient with atrial flutter and high-grade atrioventricular block: a case report
Source: Front Cardiovasc Med. 2026 Oct 7;13:1967331. doi: 10.3389/fcvm.2026.1967331 (PMC13683167; doi:10.3389/fcvm.2026.1967331)
Supplement: Supplementary file 1 [file Image1.pdf]

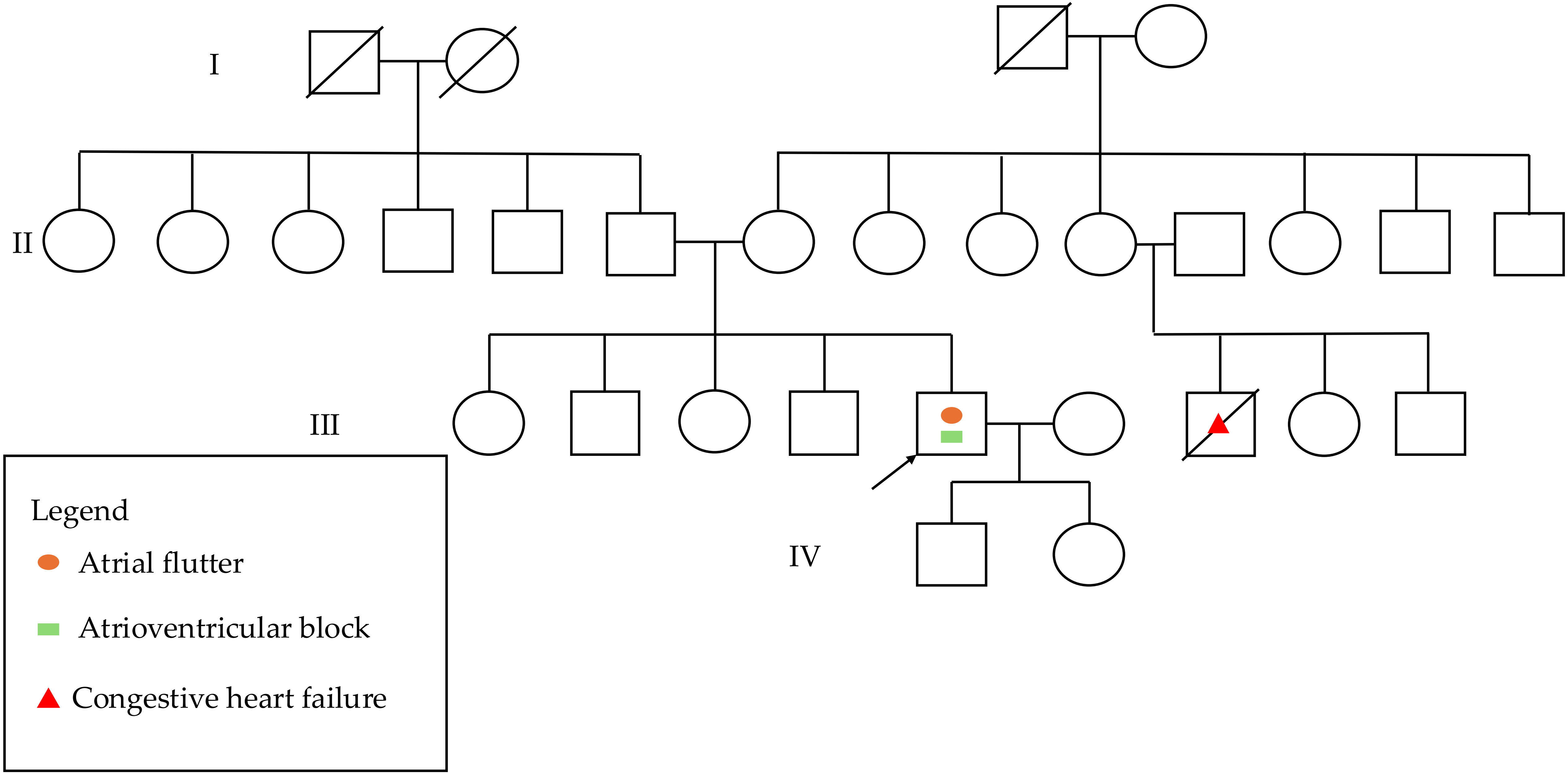
I
II
III
IV
Legend
Atrial flutter
Atrioventricular block
Congestive heart failure
